# Gestational Vitamin D Supplementation Leads to Reduced Perinatal RXRA DNA Methylation: Results From the MAVIDOS Trial

**DOI:** 10.1002/jbmr.3603

**Published:** 2019-01-18

**Authors:** Elizabeth M Curtis, Nevena Krstic, Eloïse Cook, Stefania D'Angelo, Sarah R Crozier, Rebecca J Moon, Robert Murray, Emma Garratt, Paula Costello, Jane Cleal, Brogan Ashley, Nicholas J Bishop, Stephen Kennedy, Aris T Papageorghiou, Inez Schoenmakers, Robert Fraser, Saurabh V Gandhi, Ann Prentice, M Kassim Javaid, Hazel M Inskip, Keith M Godfrey, Christopher G Bell, Karen A Lillycrop, Cyrus Cooper, Nicholas C Harvey, the MAVIDOS Trial Group

**Affiliations:** ^1^ MRC Lifecourse Epidemiology Unit University of Southampton UK; ^2^ Institute of Developmental Sciences University of Southampton UK; ^3^ Paediatric Endocrinology University Hospitals Southampton NHS Foundation Trust Southampton UK; ^4^ Academic Unit of Child Health Sheffield Children's Hospital University of Sheffield Sheffield UK; ^5^ Nuffield Department of Obstetrics and Gynaecology John Radcliffe Hospital University of Oxford Oxford UK; ^6^ MRC Elsie Widdowson Laboratory Cambridge UK; ^7^ Department of Medicine Faculty of Medicine and Health Sciences University of East Anglia Norwich UK; ^8^ Sheffield Hospitals NHS Trust (University of Sheffield) Sheffield UK; ^9^ NIHR Oxford Biomedical Research Centre University of Oxford Oxford UK; ^10^ NIHR Southampton Biomedical Research Centre University of Southampton and University Hospital Southampton NHS Foundation Trust Southampton UK

**Keywords:** EPIGENETIC, METHYLATION, RXRA, VITAMIN D, OSTEOPOROSIS, EPIDEMIOLOGY

## Abstract

We have previously demonstrated inverse associations between maternal 25(OH)‐vitamin D status and perinatal DNA methylation at the retinoid‐X‐receptor‐alpha *(RXRA)* locus and between *RXRA* methylation and offspring bone mass. In this study, we used an existing randomized trial to test the hypothesis that maternal gestational vitamin D supplementation would lead to reduced perinatal *RXRA* locus DNA methylation. The Maternal Vitamin D Osteoporosis Study (MAVIDOS) was a multicenter, double‐blind, randomized, placebo‐controlled trial of 1000 IU/day cholecalciferol or matched placebo from 14 weeks’ gestation until delivery. Umbilical cord (fetal) tissue was collected at birth and frozen at −80°C (*n* = 453). Pyrosequencing was used to undertake DNA methylation analysis at 10 CpG sites within the *RXRA* locus (identified previously). *T* tests were used to assess differences between treatment groups in methylation at the three most representative CpG sites. Overall, methylation levels were significantly lower in the umbilical cord from offspring of cholecalciferol‐supplemented mothers, reaching statistical significance at four CpG sites, represented by CpG5: mean difference in % methylation between the supplemented and placebo groups was −1.98% (95% CI, −3.65 to −0.32, *p* = 0.02). ENCODE (Encyclopedia of DNA Elements) evidence supports the functionality of this locus with strong DNase hypersensitivity and enhancer chromatin within biologically relevant cell types including osteoblasts. Enrichment of the enhancer‐related H3K4me1 histone mark is also seen in this region, as are binding sites for a range of transcription factors with roles in cell proliferation, response to stress, and growth factors. Our findings are consistent with previous observational results and provide new evidence that maternal gestational supplementation with cholecalciferol leads to altered perinatal epigenetic marking, informing mechanistic understanding of early life mechanisms related to maternal vitamin D status, epigenetic marks, and bone development. © 2018 The Authors. *Journal of Bone and Mineral Research* Published by Wiley Periodicals Inc.

## Introduction

It is becoming increasingly recognized that environmental factors acting through epigenetic mechanisms induce persistent changes in gene expression, leading to differences in phenotype.^1^, ^2^ Various examples of such epigenetic mechanisms have come from the natural world, and also from experimental animal studies. For example, altered pregnancy diet in rats has been shown to lead to modification of DNA methylation, gene expression, and phenotype in the offspring.^1^, ^2^, ^3^, ^4^ Evidence for the relevance of such mechanisms in human disease is increasing; we have recently documented associations between perinatal DNA methylation at particular loci and bone phenotype in the offspring.^5^, ^6^ We previously demonstrated that methylation at the cyclin‐dependent kinase inhibitor 2A (*CDKN2A)* 6 and retinoid‐X‐receptor‐alpha (*RXRA*)^5^ loci in umbilical cord DNA was associated with offspring bone mass in childhood in the Southampton Women's Survey (SWS) mother–offspring cohort. RXRA is an essential part of vitamin D signaling, forming a heterodimer with the vitamin D receptor in the nuclear action of 1,25(OH)_2_‐vitamin D. We reasoned that this latter observation might be of key relevance to our demonstrations of associations between maternal 25(OH)‐vitamin D status in pregnancy and offspring bone mass,^7^, ^8^, ^9^ together with our finding of a positive effect of maternal vitamin D supplementation during pregnancy on neonatal bone mass for winter births (when background 25(OH)‐vitamin D concentrations are lowest).^10^ In the SWS, methylation at one CpG site upstream of the *RXRA* promoter was associated with a marker of maternal pregnancy 25(OH)‐vitamin D status, with greater 25(OH)‐vitamin D status associated with lower *RXRA* promoter methylation.^5^ Clearly, causation cannot be concluded from an observational study; therefore, we hypothesized, in the setting of the Maternal Vitamin D Osteoporosis Study (MAVIDOS) randomized, double‐blind, placebo‐controlled trial of vitamin D supplementation in pregnancy,^10^ that this intervention would lead to reduced *RXRA* DNA methylation in umbilical cord tissue at birth compared with placebo.

## Participants and Methods

### Participants: The Maternal Vitamin D Osteoporosis Study

We analyzed *RXRA* DNA methylation data from the MAVIDOS study, a multicenter, double‐blind, randomized, placebo‐controlled trial of vitamin D supplementation in pregnancy, in which the primary outcome was neonatal bone mass. The study methods and primary findings have been published previously.^10^, ^11^ The study was approved by the Southampton and South West Hampshire Research Ethics Committee. MAVIDOS was registered prospectively (International Standard Randomised Controlled Trial Registry: ISRCTN 82927713; European Clinical Trials Database: EudraCT 2007‐001716‐23) with full approval from the UK Medicines and Healthcare Products Regulatory Agency. Written informed consent was obtained from all participants.

Women attending one of three UK hospitals (University Hospital Southampton NHS Foundation Trust, Southampton; Oxford University Hospitals NHS Foundation Trust, Oxford; Sheffield Hospitals NHS Trust, University of Sheffield, Sheffield) for early pregnancy ultrasound screening (11 to 14 weeks’ gestation) between October 6, 2008 and February 11, 2014 were invited to participate in the study.^10^, ^11^ Inclusion criteria were age over 18 years, singleton pregnancy, and gestation less than 17 weeks based on last menstrual period and ultrasound measurements. Exclusion criteria included women with known metabolic bone disease, renal stones, hyperparathyroidism or hypercalciuria, those taking medication known to interfere with fetal growth, fetal anomalies on ultrasonography, and women already using >400 IU/day vitamin D supplementation. A screening blood sample was obtained and analyzed on the local NHS platform [all three hospitals participate in the DEQAS (Vitamin D External Quality Assessment Scheme); http://www.deqas.org/]; women with 25(OH)D between 25 and 100 nmol/L and serum calcium <2.75 mmol/L were eligible to enroll in the study.

Participants were randomized to receive either cholecalciferol 1000 IU/day or matched placebo [Merck KGaA, Darmstadt, Germany)/Sharp Clinical Services (previously DHP‐Bilcare), Crickhowell, UK], from before 17 weeks’ gestation until delivery. Packs of study treatment were randomly assigned in a 1:1 ratio by Sharp Clinical Services using a computer‐generated sequence in randomly permuted blocks of 10, starting randomly midway through the block, and sequentially numbered, before delivery to the study sites, and then were dispensed in order by each study pharmacist. The study medication was provided in a blister pack in a single box containing all medication for the whole pregnancy. The participants, those providing antenatal and intrapartum care, and all field researchers involved in data collection and sample analysis were blinded to the intervention. All participants received standard antenatal care, and could continue self‐administration of dietary supplements containing up to 400 IU/day vitamin D.

### Maternal assessments during pregnancy

The participants attended the research center for a detailed assessment of diet (including supplement use), lifestyle (smoking, physical activity participation, employment), and health (past medical history, current medication use) using interviewer‐led questionnaires both prior to commencing the study medication, and again at 34‐weeks’ gestation. Ethnicity was reported by the participant and categorized as white or non‐white.

### Assessment of 25(OH)D status

A nonfasted venous blood sample was obtained on the day that the study medication was dispensed and also at 34‐weeks’ gestation; serum was stored at −80°C. 25(OH)D was assessed by chemiluminescent assay (liaison automated platform; DiaSorin, Stillwater, MN, USA). All samples were analyzed in a single batch at the end of the study at MRC Human Nutrition Research, Cambridge, UK. Details of assay performance and quality control through participation in the DEQAS, US National Institute of Standards and Technology (NIST), and the UK National External Quality Assessment Service (NEQAS) are given elsewhere.^12^, ^13^

### Neonatal DXA

All neonates underwent DXA assessment at whole‐body minus head and lumbar spine sites (Hologic Discovery, Hologic Inc, Bedford, MA, USA, or GE‐Lunar iDXA, GE‐Lunar, Madison, WI, USA, with neonatal software) within 2 weeks of birth. The current analysis uses the whole‐body minus head measures. The infant was undressed, clothed in a standard towel, fed, and pacified before the assessment. Each instrument underwent daily quality control with cross‐calibration between sites. The total radiation dose was estimated to be 0 · 04 mSv, equivalent to about 7‐days’ exposure to background radiation in the UK. All DXA images were reviewed for movement artifacts and quality by two operators (NCH and RJM), who were blinded to treatment allocation.

### Umbilical cord DNA extraction

Immediately following delivery, a 5‐ to 10‐cm segment was cut from the midportion of each cord, flushed with saline to remove fetal blood, flash‐frozen in liquid nitrogen, and stored at −80°C until required for DNA isolation. Genomic DNA was isolated from frozen archived umbilical cord tissue by classical proteinase K digestion and phenol:chloroform extraction.

### Quantitative DNA methylation analysis and pyrosequencing

The region of interest is in close proximity of the *RXRA* gene locus, 2252 base pairs upstream from the transcriptional start site. It contains 12 CpG dinucleotides (chr9: 137215735 to 137216064, human genome hg19/GRCh37 build; Supplementary Fig.  1). We used sodium bisulfite targeted pyrosequencing (Pyromark MD; QIAGEN, Hilden, Germany; https://www.qiagen.com/fi/resources/technologies/pyrosequencing-resource-center/technology-overview/)^14^ to carry out in‐depth analysis of the methylation status of 10 out of 12 CpGs within the previously identified differentially methylated region of *RXRA* in umbilical cords. Pyrosequencing was not performed on CpG 6 and CpG 7 [at genomic coordinates (hg19) chr9 137215867 and 137215956, respectively] for sample conservation purposes because of their distance from other CpGs (therefore requiring separate amplicons), as shown in Supplementary Fig.  1. Inter‐ and intraplate controls were added to each plate as a control for inter‐ and intraplate variability, and 0% and 100% methylation controls were run to ensure that the full range of methylation could be detected. The genomic coordinates for the *RXRA* CpG sites are provided in Supplementary Table  1.

### Statistical analysis

Women who had delivered a liveborn infant and babies who had umbilical cord *RXRA* pyrosequencing analysis were included in the analysis. All outcomes were assessed for normality via visual inspection of histograms. Percentage DNA methylation at all *RXRA* CpGs analyzed was normally distributed, except at CpG 1 and CpG 3. Characteristics of the women in the two treatment arms were compared using *t* tests, and Mann‐Whitney *U* and χ^2^ tests for normally distributed, non‐normally distributed, and categorical variables, respectively. Characteristics of the MAVIDOS babies (boys versus girls) for whom neonatal DXA and *RXRA* methylation data were available were also compared. Neonatal DXA indices were whole‐body minus head bone area (BA), BMC, and areal bone mineral density (aBMD). Continuous child characteristics were summarized using mean (SD) or median (interquartile range [IQR]). Categorical variables were summarized using percentages. Differences in continuous variables between boys and girls were tested using *t* tests or Mann‐Whitney *U* tests where appropriate. All participants were analyzed by the group to which they were originally randomized. Differences in *RXRA* DNA methylation between the two treatment groups were compared using *t* tests or Mann‐Whitney *U* tests for normally distributed and non‐normally distributed variables, respectively. *RXRA* methylation was Fisher‐Yates transformed to SDs. Separate linear regression analyses were carried out to analyze the difference in methylation between the treatment groups. We analyzed the interaction between treatment group, *RXRA* methylation, and season of birth because of previously described seasonal variations in 25(OH)D concentrations reported in many previous studies. To ensure adequate sample sizes, we defined season of birth as a binary variable using the UK Meteorological Office classification, combining winter (December to February) with spring (March to May) to give an overall “winter” variable (December to May), and summer (June to August) with autumn (September to November) to give an overall “summer” variable (June to November). To explore associations between *RXRA* methylation and bone outcomes, linear regression analyses were carried out, adjusted for treatment group and sex where appropriate.

Based on previous findings, we recognized that there was likely to be colinearity between the individual exposures and outcomes,^5^, ^6^ so we undertook a data reduction approach by investigating clustering of the CpG methylation.^6^ Our approach was appropriate given the relatively small number of tests in our analysis, compared with larger scale genome‐wide associations studies, for which methods such as Bonferroni or the Benjamini‐Hochberg/false discovery rate corrections for multiple testing would be appropriate.^15^ Previous studies have shown that where clusters of differential CpGs can be identified, they are more likely to be of functional relevance than are individual CpG changes.^16^ By investigating the correlation between methylation at each of the individual CpG sites (Supplementary Table  2) and calculating the median absolute deviation (MAD) from the median for each site (Supplementary Table  3), we grouped the CpG sites into three clusters (CpG 1 to CpG 5, CpG 8 to CpG 11, CpG 12), with each cluster represented by the site with the highest MAD score (ie, the site with the greatest variability within the cluster): CpG sites 5, 11, and 12, respectively. For completeness, we also used the Simes’ modification of the Bonferroni method to undertake a *p*‐value correction on the analyses, using the Stata “qqvalue” command (StataCorp, College Station, TX, USA), which is similar to the “p.adjust” command in R https://www.rdocumentation.org/packages/stats/versions/3.1.1/topics/p.adjust. These are presented as *q* values in the relevant results Tables 2, 3 and Supplementary Table  4.

Further biological support for this clustering was provided by exploration of the ENCODE data,^17^ demonstrating distinct DNase I hypersensitivity sites at either end of the differentially methylated region, and discrete grouping of transcription factor binding. All analyses were performed in Stata v14 (StataCorp). A *p* value of <0.05 was considered statistically significant.

## Results

### Characteristics of participants

**Figure 1:**
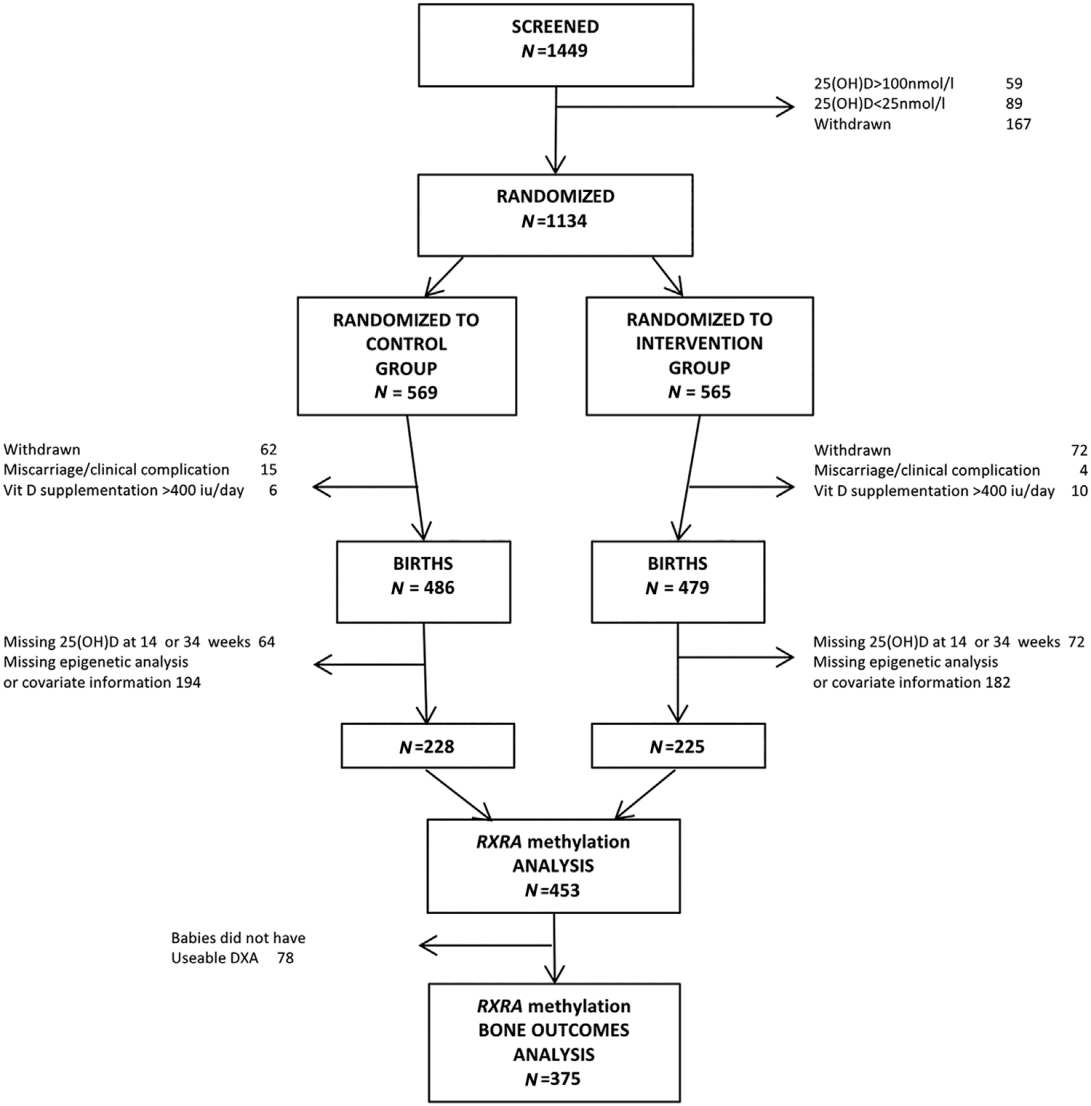
MAVIDOS trial consort diagram.

There were 965 women (85%) who remained in the study until delivery (Fig. 1).^10^ There were 486 live births in the control group and 479 in the cholecalciferol group, of which 228 and 225 umbilical cords, respectively, underwent pyrosequencing of the *RXRA* region of interest. Seventy‐eight babies for whom pyrosequencing results were available did not have a useable DXA scan (43 randomized to placebo, 35 to cholecalciferol), leaving 375 babies with *RXRA* methylation analyses, DXA outcomes, and the relevant maternal information. Of the 453 women included in the initial analysis, the mean age at delivery was 30.9 (SD 5.2) years in the placebo group and 30.7 (SD 5.1) years in the cholecalciferol supplemented group. Baseline characteristics of women in the placebo and cholecalciferol groups at randomization were similar (Table 1A). Neonatal DXA whole‐body minus head bone measurements were available for 202 boys (91 born to mothers randomized to placebo, 111 to cholecalciferol) and 173 girls (94 born to mothers randomized to placebo, 79 to cholecalciferol; Table 1B). A χ^2^ test demonstrated no difference in the sex distribution of the treatment groups. There was no difference in gestational age between the boys and girls. As would be expected, boys had a greater average whole‐body minus head bone mineral content (g) and bone area (cm^2^) than girls. In this subset of the MAVIDOS trial population, no differences in gestational age or whole‐body minus head DXA outcomes were observed in the babies by maternal randomization group to placebo or 1000 IU cholecalciferol (Table 1C).

**Table 1A:** Baseline Characteristics of the Randomly Assigned Pregnant Women Included in the Analysis

	*n*	*Placebo (N = 228)*	*Cholecalciferol 1000 IU/day (N = 225)*
*Mean (SD) or median (IQR)*			
*Age (years)*	427	30.9 (5.2)	30.7 (5.1)
*Height (cm)*	423	166.4 (6.3)	165.3 (6.1)
*Weight (kg)*	427	73.6 (13.1)	71.6 (14.1)
*Pregnancy weight gain (kg)*	415	9.4 (3.6)	9.7 (3.5)
*BMI (kg/m^2^)†*	423	25.7 (23.0,29.7)	24.9 (22.4,28.8)
*Sum of skinfold thickness (mm)*	360	81.9 (27.0)	78.3 (29.1)
*25(OH)D at 14 weeks (nmol/L)*	445	45.1 (16.2)	44.4 (15.2)
*25(OH)D at 34 weeks (nmol/L)*	432	42.8 (20.0)	66.3 (19.8)
*n (%)*			
*Nulliparous*	427	99 (46.3)	91 (42.7)
*Educational qualification > A level*	423	156 (74.3)	163 (76.5)
*Current smoker*	426	16 (7.5)	12 (5.7)
*Strenuous exercise ≥ once a week*	390	22 (11.3)	32 (16.4)

Values are *n* (%), mean (SD), or median (interquartile range [IQR]). P difference 25(OH)D at 34 weeks, cholecalciferol supplemented versus placebo group, *p* < 0.001.

**Table 1B:** Whole‐Body Minus Head DXA Characteristics of the MAVIDOS Babies by Sex for Whom DXA and RXRA Methylation Data Are Available

	Boys (*n* = 202)	Girls (*n* = 173)	*p* difference between boys and girls
Gestational age (weeks)	40.2 (1.3)	40.1 (1.4)	0.22
BA (cm^2^)	306.5 (34.8)	295.8 (33.8)	0.003
BMC (g)	63.1 (10.7)	59.9 (10.5)	0.004
aBMD (g/cm^2^)	0.205 (0.018)	0.202 (0.020)	0.100

*p* values <0.05 are in bold.

BA = bone area; BMC = bone mineral content; aBMD = areal bone mineral density.

**Table 1C:** Whole‐Body Minus Head DXA Characteristics of the MAVIDOS Babies by Maternal Randomization Group for Whom DXA and *RXRA* Methylation Data Are Available

	Placebo (*N* = 185)	Cholecalciferol 1000 IU/day (*N* = 190)	*p* difference between maternal randomization group
Gestational age (weeks)	40.1 (1.4)	40.2 (1.2)	0.23
BA (cm^2^)	299.4 (36.7)	303.6 (32.6)	0.24
BMC (g)	61.2 (10.9)	62.0 (10.6)	0.46
aBMD (g/cm^2^)	0.204 (0.019)	0.203 (0.019)	0.93

*p* values <0.05 are in bold.

BA = bone area; BMC = bone mineral content; aBMD =  areal bone mineral density.

### Cholecalciferol supplementation and perinatal RXRA methylation

**Figure 2:**
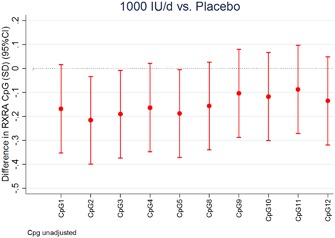
Difference in *RXRA* DNA methylation at each CpG site between cholecalciferol 1000 IU/day supplemented group and placebo group (expressed as SDs). Each bar is the outcome of a separate linear regression (mean difference and 95% CI).

Percentage methylation at the *RXRA* differentially methylated region (DMR) varied greatly across the 10 CpG sites measured, for example, ranging from 29.0% to 81.4% at CpG 5 (mean 47.7%, SD 9.0%; Supplementary Table  1). However, percentage methylation tended to be lower in the cholecalciferol‐supplemented group than in the placebo group (Fig. 2). At CpG 5 (representing CpG 1 to CpG 5), mean (SD) percentage methylation was 46.7% (8.2%) in the cholecalciferol group and 48.7% (9.7%) in the placebo group (mean difference −1.98 percentage points, *p* = 0.02). Although percentage methylation at both CpG 11 (representing CpG 8 to CpG 11) and CpG 12 was lower in cholecalciferol than placebo group births, these differences were not statistically significant (Table 2 and Supplementary Table  4). We observed no consistent associations between maternal 25(OH)D status at 34 weeks, or change in 25(OH)D from early to late pregnancy, and RXRA methylation in umbilical cord tissue across the cohort. However, there was evidence of an interaction between change in 25(OH)D between 14‐ and 34‐weeks’ gestation 25(OH)D and treatment allocation to cholecalciferol or placebo on RXRA methylation at CpG 11 (*p* = 0.022).

**Table 2:** RXRA DNA Methylation in Cholecalciferol 1000 IU/day Supplemented and Placebo Groups

	*n*	*% methylation Cholecalciferol 1000 IU/day*	*% methylation Placebo*	*Mean diff. % methylation*	*95% CI*	*p difference*	*q difference*
RXRA CpG 5	447	46.7 (8.2)	48.7 (9.7)	**−1.98**	**−3.65, −0.32**	**0.02**	**0.06**
RXRA CpG 11	446	58.3 (7.5)	58.9 (8.1)	−0.67	−2.12, 0.78	0.36	0.36
RXRA CpG 12	446	66.1 (5.8)	66.9 (6.6)	−0.84	−1.99, 0.31	0.15	0.225

CpG 5 represents CpG 1 to CpG 5; CpG 11 represents CpG 8 to CpG 11. *p* values < 0.05 are in bold. q values were obtained using the Simes method. Difference in methylation = mean (cholecalciferol 1000 IU/day) – mean (placebo).

### Interactions between season of birth, treatment group, and percentage DNA methylation at *RXRA*

Greater increases in maternal 25(OH)D status were seen in summer (June to November) than in winter (December to May) births; the increase in 25(OH)D during pregnancy was more than double in the women giving birth in summer. In summer births, mean (SD) change in 25(OH)D between 14‐ and 34‐weeks’ gestation was 8.1 (16.0) nmol/L in the placebo group (*n* = 124), and 28.0 (19.8) nmol/L in the vitamin D supplemented group (*n* = 127). In winter births, mean (SD) change in 25(OH)D between 14‐ and 34‐weeks’ gestation was −15.0 (17.6) nmol/L in the placebo group (*n* = 103), and 13.6 (22.6) nmol/L in the vitamin D supplemented group (*n* = 95).

There was evidence of statistically significant interactions for the outcome of *RXRA* methylation between treatment allocation (cholecalciferol versus placebo) and season of birth (at all three representative CpGs: CpG 5, *p* = 0.02; CpG 11, *p* = 0.009; and CpG 12 *p* = 0.01). The effect of treatment group on *RXRA* methylation appeared greater in summer than winter births (Table 3). In summer births, there was a difference in percentage methylation at *RXRA* CpG 5, CpG 11, and CpG 12 between treatment groups ranging from −3.69% at CpG 5 (*p* = 0.001), to −2.38% at CpG 11 (*p* = 0.01), and −2.13% at CpG 12 (*p* = 0.005), but the differences between groups were nonsignificant for winter births. This interaction persisted after adjustment for potential differences in maternal characteristics between the season groups (maternal BMI and skinfold thickness), and for other factors known to influence methylation (offspring sex and maternal smoking).

**Table 3:** RXRA DNA Methylation in Cholecalciferol 1000 IU/day Supplemented and Placebo Groups, Stratified by Season: Winter Births (December to May) and Summer Births (June to November)

	*Winter births (Dec to May)*				*Summer births (June to Nov)*			
*CpG*	Mean diff. % methylation	95% CI	*p*	q	Mean diff. % methylation	95% CI	*p*	q
*RXRA CpG 5*	0.27	(−2.27, 2.82)	0.83	0.83	**−3.69**	**(−5.92,−1.45)**	**0.001**	**0.004**
*RXRA CpG 11*	1.51	(−0.73, 3.76)	0.18	0.55	**−2.38**	**(−4.29,−0.47)**	**0.02**	**0.01**
*RXRA CpG 12*	0.79	(−1.00, 2.58)	0.38	0.58	**−2.13**	**(−3.60,−0.65)**	**0.005**	**0.007**

Difference in methylation = mean (cholecalciferol 1000 IU/day) – mean (placebo). *p* values <0.05 are in bold. q values were obtained using the Simes method.

### *RXRA* methylation and offspring bone indices measured by DXA

**Figure 3:**
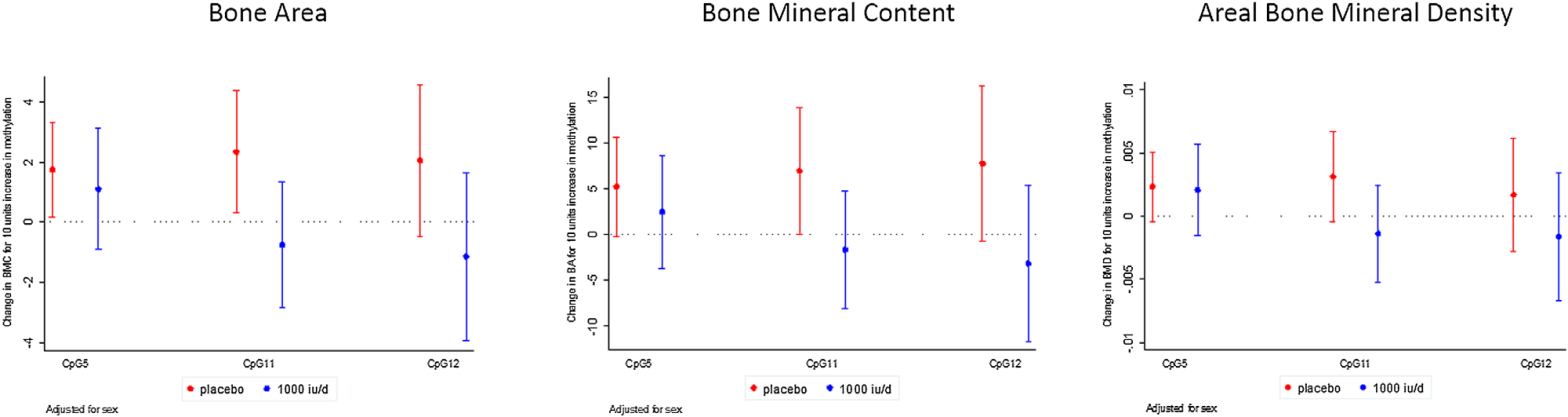
Associations between *RXRA* methylation at CpG 5, CpG 11, and CpG 12 and whole‐body minus head bone area (cm^2^), bone mineral content (g), BMD (g/cm^2^), adjusted for sex, by treatment group (placebo [red bars] or 1000 IU cholecalciferol daily [blue bars]). Outcomes expressed per 10% increase in methylation.

In the population as a whole, there were modest positive associations between *RXRA* methylation at CpG 5 and offspring whole‐body minus head BA, BMC, and aBMD (Table 4). However, on stratification according to treatment allocation, associations were noted in the placebo, but not the cholecalciferol supplemented groups, as documented in Fig. 3. In the placebo group (red bars in Fig. 3), *RXRA* methylation at CpG 11 was positively associated with BA (β = 6.96 cm^2^ per 10% increase in methylation, *p* = 0.05). There was also a tendency towards positive associations between methylation and BA at CpG 5 and CpG 12. Furthermore, again in the placebo group, methylation at CpG 5 and CpG 11 was positively associated with offspring BMC (at CpG 5, β = 1.75g per 10% increase in methylation, *p* = 0.03; at CpG 11, β = 2.34g per 10% increase in methylation, *p* = 0.02). Conversely, in the cholecalciferol‐supplemented group (blue bars in Fig. 3), no statistically significant associations were found between methylation at CpG 5, CpG 11, and CpG 12 and offspring neonatal DXA bone outcomes (Supplementary Table  5).

**Table 4:** Relationships Between Perinatal Methylation in Umbilical Cord at CpG Sites Within the RXRA Region of Interest and Bone Indices at Birth (Measured by DXA, Whole Body Minus Head)

	BA, (cm^2^)		BMC, (g)		aBMD, (g/cm^2^ )	
RXRA CpG	β (95% CI)	*p*	β (95% CI)	*p*	β (95% CI)	*p*
CpG 5	**4.18 (0.16, 8.20)**	**0.04**	**1.50 (0.27, 2.74)**	**0.02**	**0.002 (0.000, 0.004)**	**0.05**
CpG 11	2.77 (−1.93, 7.48)	0.25	0.84 (−0.61, 2.30)	0.26	0.001 (−0.002, 0.004)	0.47
CpG 12	2.85 (−3.19, 8.88)	0.35	0.62 (−1.25, 2.49)	0.52	0.000 (−0.003, 0.003)	0.91

Associations are adjusted for sex and treatment group. β coefficients and 95% CIs have been multiplied by 10 and therefore represent the change associated with a 10% increase in methylation. *p* values <0.05 are in bold.

BA = bone area; BMC = bone mineral content; aBMD = areal bone mineral density.

### ENCODE functional analysis

The DMR itself resides within the upstream CpG island shore region (within 2 kb) of the *RXRA* 5'CpG island. ENCODE consortium data were interrogated for functional evidence within this location.^17^ This investigation revealed that the region of interest within the *RXRA* locus contains a cluster of DNase I hypersensitive sites (DHS, a general regulatory marker, often found within regulatory elements such as promoters and enhancers^18^), identified in 84 cell lines out of 125 (see Supplementary Fig.  2, with examples from the chorion and osteoblast cell lines highlighted). Furthermore, significant enrichment of the enhancer‐related H3K4me1 histone mark across a range of tissue types was found both across and within 250 bp of the DMR (Supplementary Fig.  2). Enhancer loci may show dynamic DNA methylation indicative of transcription factor interaction within these functional regions.^19^ Consistent with this, genome segmentations from ENCODE (displaying chromatin state segmentations from six cell lines) predict weak enhancer activity or an open chromatin *cis* regulatory element at the *RXRA* DMR (yellow region in Supplementary Fig.  2) at the *RXRA* DMR. Finally, ENCODE transcription factor binding data demonstrate significant binding within the *RXRA* DMR. The numbers of transcription factor binding sites found at the *RXRA* DMR in the ENCODE database vary between cell types. For example, in the cell line, MCF‐7, which is highly responsive to estrogen and TSH,^20^ three transcription factors bind with high affinity at the *RXRA* DMR (MYC, CTCF, and POL2RA). In summary, these findings suggest the *RXRA* DMR as a region of significant functional activity across a range of cell types, with evidence of strong DNAse I hypersensitivity sites, weak enhancer or *cis* regulatory element activity, and transcription factor binding.

### *RXRA* methylation and gene expression in vitamin D‐treated placental villous fragments

To experimentally investigate the influence of vitamin D on *RXRA* methylation in perinatal tissue (placenta), we collected six placentas from healthy term pregnancies, outside the MAVIDOS trial (with full ethics approval, REC 11/sc/0323), within 30 min of delivery. Placental villous fragments were cultured in buffered solution with or without 20 μM 25‐hydroxyvitamin D [25(OH)D] (for detailed methods, see Supplementary Material). The placental samples were snap frozen and stored at −80°C. DNA was extracted, and DNA methylation was measured using the Illumina EPIC 850k array (Illumina, San Diego, CA, USA). CpGs in which DNA methylation was altered were identified using a Wilcoxon signed‐rank test. RNA was also extracted, and stranded RNA sequencing was performed; differentially expressed genes following 25(OH)D treatment were identified. In human placental villous fragments, 25(OH)D treatment altered DNA methylation at six CpG sites in the *RXRA* gene: decreased at four CpG sites (‐0.80% to −2.67%, *p* = 0.04 and *p* = 0.02, respectively) and increased in two (1.10% to 1.41%, *p* = 0.04 and *p* = 0.01, respectively), as shown in Supplementary Table  6. Through RNA sequencing, RXRA gene expression was shown to increase following 25(OH)D treatment (log fold change 0.50, *p* = 0.04).

## Discussion

In this study we have demonstrated, to our knowledge for the first time in a randomized controlled trial setting, that supplementation with cholecalciferol in pregnancy is associated with reduced methylation at specific regions near to the *RXRA* promoter in fetal DNA derived from the umbilical cord of the offspring. Percentage methylation levels measured by pyrosequencing were lower in the cholecalciferol supplemented group than the placebo group (statistically significantly at the cluster of CpG sites represented by CpG 5), raising the possibility of site‐specificity for a molecular interaction between 25(OH)D in pregnancy and DNA methylation.^21^

These results are consistent with our previous observational findings in the SWS, in which a negative association was found between an estimate of maternal‐free 25(OH)‐vitamin D and *RXRA* methylation at CpG 4/5,^5^ measured using the Sequenom MassARRAY EpiTYPER (Sequenom Laboratories, San Diego, CA, USA). Additionally, the associations between *RXRA* methylation and neonatal bone indices in the placebo group replicated those observed previously in the SWS; conversely, in the present study, the direction of the association appeared to be reversed (albeit not reaching statistical significance) in the group whose mothers were supplemented with cholecalciferol. It is interesting that the methylation difference between treatment and placebo groups in the present study was greater in summer than winter births. The increase in 25(OH)D from baseline to 34 weeks was markedly greater for summer than winter deliveries, although the absolute difference between groups at 34 weeks was marginally less in summer than in winter, suggesting that greater increases in 25(OH)D across pregnancy might facilitate methylation differences consequent to vitamin D supplementation. However, given that RXRA interacts with several different nuclear hormone receptors, such as thyroid hormone receptor and PPAR‐gamma, activation of either of which tends to have detrimental effects on bone, it is possible that we are seeing the net result of a complex series of interrelationships at this molecular level, with exogenous vitamin D perhaps modifying the balance in RXRA interaction between receptor types, resulting in heterogeneous associations between *RXRA* methylation and bone indices. Such considerations may be relevant both to the skeletal and to the seasonal differences we observed, although ultimately these questions must remain the focus of future research. Interestingly, we observed no consistent associations between maternal 25(OH)D in late pregnancy and *RXRA* methylation, but these measures were in different tissues, 6 weeks apart, and we were able to directly test whether treatment of perinatal tissue (placenta) with vitamin D would alter *RXRA* methylation. Thus, consistent with the findings from the MAVIDOS trial, in a small study of human placental villous fragments *RXRA* methylation appeared to be lowered at several CpG sites by the addition of 25(OH)D, and indeed *RXRA* expression upregulated overall, suggesting a specific role for the vitamin D–*RXRA* interaction.

Although the exact nature of the mechanistic underpinnings of our findings remains to be elucidated, there are several routes by which maternal 25(OH)D status might influence perinatal *RXRA* methylation. As previously stated, RXRA forms a heterodimer with several nuclear hormones known to influence bone metabolism, including 1,25(OH)_2_‐vitamin D, perhaps implying that maternal 25(OH)D status plays a permissive role in the transcriptional regulation of the *RXRA* gene. Studies have shown that vitamin D may interact with the epigenome on multiple levels,^17^, ^22^, ^23^, ^24^, ^25^ and our evaluation of public data from ENCODE suggests that methylation at the studied CpG sites is likely to have functional relevance, with evidence for DNase I hypersensitive regions, enhancer activity, and transcription factor binding. Furthermore, this suggested function within the DMR, which itself resides within the shore region of the 5’ CpG island. This location has been associated with influence on gene expression.^26^ Epigenome‐wide association studies (EWASs) have also provided some insight into the actions of vitamin D on DNA methylation. A small EWAS of DNA methylation in severely vitamin‐D‐deficient African‐American adolescents demonstrated associations between vitamin D status and methylation in several genes, including genes involved in vitamin D metabolism such as the 24 and 25‐hydroxylase genes. In the context of low serum vitamin D levels, the promoter of *CYP2R1* may become methylated; this is reversible on exposure to vitamin D.^27^ Other studies have assessed the DNA methylation in CYP enzymes, which are part of the vitamin D metabolism pathway, and found a relationship between methylation of the genes *CYP2R1* (25‐hydroxylase) and *CYP24A1* (24‐hydroxylase) and variations in circulating 25(OH)D levels.^28^ However, a study using the ALSPAC (Avon Longitudinal Study of Parents and Children) cohort and the Norwegian Mother and Child Cohort (MoBa) in which maternal 25(OH)D was measured in midpregnancy, demonstrated no convincing associations between maternal 25(OH)D status and DNA methylation in the umbilical cord blood (as opposed to umbilical cord tissue in our study) of 1416 newborn babies using Illumina 450k DNA methylation array analysis, thereby covering 473,731 CpG DNA methylation sites.^29^ The authors suggested that to further identify associations, larger consortium studies, expanded genomic coverage, and the investigation of alternative cell types or 25(OH)D status at different gestational time points might be needed.

The data presented are from a placebo‐controlled, double‐blind, randomized trial, using the gold standards of pyrosequencing to determine CpG site‐specific DNA methylation and DXA to assess bone mass. However, the limitations of our study must be considered. First, we have analyzed methylation in cells from whole umbilical cord; therefore, it is possible that the differential methylation we observed arose from different component cells in individual samples (eg, fibroblasts and epithelial cells). The difference in DNA methylation between treatment and control groups may thus partly reflect different proportions of cells and their cell‐specific DNA methylation. However, any unaccounted cell heterogeneity may represent proportional differences that are related to the observed phenotypic outcomes,^30^, ^31^ and so potentially on the causal pathway. Second, owing to stipulations made during the ethics approval process, participants with baseline 25(OH)D concentrations less than 25 nmol/L or greater than 100 nmol/L could not be included. In addition, the study population did not include many women who were of nonwhite ethnicity, which again would affect the generalizability of our findings to multiethnic populations. Third, DXA assessment in neonates presents some difficulties, including the low absolute BMC of newborn babies and their tendency to move. However, the validity of DXA in small animals, of comparable size to neonates, has been documented^32^ and appropriate DXA software was used. Fourth, some participants were taking vitamin D supplements in addition to the study drug, though supplement use was recorded at interview and did not differ between the treatment groups. Fifth, though we have previously excluded the presence of any SNPs at the CpG sites of interest at the *RXRA* locus by sequencing, we did not have information permitting exclusion of a genetic *cis* or *trans*‐effect of local or distant SNPs, respectively. These could influence either associations between vitamin D supplementation and *RXRA* methylation, or influence both *RXRA* methylation and the child's bone phenotype. Sixth, we did not have measurements of 25(OH)D in umbilical cord blood; thus we were not able to directly assess a potential mediating role for 25(OH)D for *RXRA* methylation in the same organ at the same time. Finally, it should be noted that the analysis is post hoc and that methylation outcomes were not prespecified in the original analysis plan, and so will require replication in further intervention studies.

In conclusion, we have shown in a randomized controlled trial that maternal supplementation with cholecalciferol from 14‐weeks’ gestation to delivery leads to lower levels of DNA methylation at the *RXRA* promoter in umbilical cord. This informs our understanding of early life mechanisms underpinning maternal vitamin D status, epigenetic change, and bone development, and may suggest a novel biomarker for a child's future bone health.

## Disclosures

CC reports personal fees from ABBH, Amgen, Eli Lilly, GSK, Medtronic, Merck, Novartis, Pfizer, Roche, Servier and Takeda, outside the submitted work. NCH reports personal fees, consultancy, lecture fees and honoraria from Alliance for Better Bone Health, AMGEN, MSD, Eli Lilly, Servier, Shire, Radius Health, UCB, Consilient Healthcare and Internis Pharma, outside the submitted work. NJB reports remuneration from Internis Pharmaceuticals Ltd, outside the submitted work. ATP reports grants from Arthritis Research Council, during the conduct of the study. KMG reports reimbursement for speaking at Nestle Nutrition Institute conferences, grants from Abbott Nutrition & Nestec, outside the submitted work; in addition, KMG has a patent Phenotype Prediction pending, a patent Predictive Use of CpG Methylation pending, and a patent Maternal Nutrition Composition pending, not directly related to this work. HMI reports grants from Medical Research Council, Arthritis Research UK, European Union's Seventh Framework Programme, during the conduct of the study; and while not directly receiving funding from other bodies, members of her team have received funding from the following companies from other work: Danone, Nestec, Abbott Nutrition. MKJ reports personal fees from Stirling Anglia, Consilient Health and Internis, outside the submitted work. All other authors have no disclosures.

## Supporting information

Supporting Tables S1.
