# Supplementary material for: Gestational Vitamin D Supplementation Leads to Reduced Perinatal RXRA DNA Methylation: Results From the MAVIDOS Trial
Source: J Bone Miner Res. 2019 Jan 18;34(2):231–40. doi: 10.1002/jbmr.3603 (PMC6372078; doi:10.1002/jbmr.3603)
Supplement: Supplementary file 1 — Supporting Tables S1. [file JBMR-34-231-s001.docx]

**Gestational vitamin D supplementation leads to altered perinatal *RXRA* promoter DNA methylation: results from the MAVIDOS trial**

Elizabeth M Curtis^1*^, Nevena Krstic^2*^, Eloïse Cook^2*^, Stefania D’Angelo^1^, Sarah R Crozier^1^, Rebecca J Moon^1,3^, Robert Murray^2^, Emma Garratt^2^, Paula Costello^2^, Jane Cleal^2^, Brogan Ashley^2^, Nicholas J Bishop^4^, Stephen Kennedy^5^, Aris T Papageorghiou^5^, Inez Schoenmakers^6,7^, Robert Fraser^8^, Saurabh V Gandhi^8^, Ann Prentice^6^, M Kassim Javaid^9,^ Hazel M Inskip^1,10^, Keith M Godfrey^1,10^, Christopher G Bell^1,2^, Karen A Lillycrop^2+^, Cyrus Cooper^1,9,10+^, Nicholas C Harvey^1,10+^ and the MAVIDOS Trial Group

^1^MRC Lifecourse Epidemiology Unit, University of Southampton, UK; ^2^Institute of Developmental Sciences, University of Southampton, UK

^3^Paediatric Endocrinology, University Hospitals Southampton NHS Foundation Trust, Southampton, UK; ^4^Academic Unit of Child Health, Sheffield Children’s Hospital, University of Sheffield, Sheffield, UK; ^5^Nuffield Department of Obstetrics and Gynaecology, John Radcliffe Hospital, University of Oxford, Oxford, UK; ^6^MRC Elsie Widdowson Laboratory, Cambridge, UK; ^7^Department of Medicine, Faculty of Medicine and Health Sciences, University of East Anglia, Norwich, UK; ^8^Sheffield Hospitals NHS Trust (University of Sheffield), Sheffield, UK; ^9^NIHR Oxford Biomedical Research Centre, University of Oxford, Oxford, UK; ^10^NIHR Southampton Biomedical Research Centre, University of Southampton and University Hospital Southampton NHS Foundation Trust, Southampton, UK, UK. *EMC, NK and EC are joint first authors; ^+^KAL, CC and NCH are joint senior authors

MAVIDOS Trial Group: Nigel K Arden, Andrew Carr, Michael Clynes, Elaine M Dennison, Richard Eastell, M Zulf Mughal, David M Reid, Sian M Robinson

**Online Supplementary Material**

**Online Supplementary Table 1:** Percentage DNA methylation at *RXRA* in umbilical cord tissue of offspring. DMR: Chromosome 9: 137215735- 137216064, Human genome hg19/GRCh37 build (Human genome hg19/GRCh37 build). Pyrosequencing was not performed at CpG 6 and 7.

| **CpG position** | **Distance from transcriptional start site (bases)** | **Human genome 19 coordinates** | **N** | **Min (25^th^, 50^th^, 75^th^ percentile) Max** | **Mean (SD)** |
| --- | --- | --- | --- | --- | --- |
| 1 | -2686 | 137215735 | 446 | 16.9 (27.5, 31.7, 38.6) 70.6 | 34.0 (9.7) |
| 2 | -2682 | 137215739 | 449 | 37.2 (50.0, 54.9, 59.5) 89.5 | 55.5 (8.0) |
| 3 | -2673 | 137215748 | 450 | 21.0 (30.3, 34.8, 41.4) 70.0 | 36.6 (9.1) |
| 4 | -2649 | 137215772 | 444 | 32.5 (44.7, 49.3, 55.2) 80.4 | 50.7 (8.7) |
| 5 | -2642 | 137215779 | 447 | 29.0 (41.5, 46.5, 52.5) 81.4 | 47.7 (9.0) |
| 6 | -2554 | 137215867 |  |  |  |
| 7 | -2465 | 137215956 |  |  |  |
| 8 | -2406 | 137216015 | 449 | 44.6 (58.9, 63.0, 67.2) 84.5 | 63.1 (6.2) |
| 9 | -2391 | 137216030 | 448 | 29.4 (46.4, 50.5, 55.6) 80.6 | 51.5 (8.0) |
| 10 | -2387 | 137216034 | 447 | 46.9 (60.7, 64.7, 69.6) 88.9 | 65.5 (7.1) |
| 11 | -2385 | 137216036 | 446 | 37.8 (53.6, 57.5, 63.4) 84.9 | 58.6 (7.8) |
| 12 | -2357 | 137216064 | 446 | 46.1 (62.5, 66.1, 70.7) 86.1 | 66.5 (6.2) |

**Online Supplementary Table 2:** CpG Clustering. Pearson’s / Spearman’s correlation of methylation levels at CpGs 1-9 within the *RXRA* region. 3 distinct clusters are defined. (A) Correlations in the MAVIDOS trial (dark green r≥0.90, pale green r≥0.80, yellow r≥0.70, orange r≥0.60, red r≥0.50). (B) Median absolute deviation (MAD) scores within the 3 clusters in MAVIDOS trial.

|  | *RXRA* CpG 1† | *RXRA* CpG 2 | *RXRA* CpG 3† | *RXRA* CpG 4 | *RXRA* CpG 5 | *RXRA* CpG 8 | *RXRA* CpG 9 | *RXRA* CpG 10 | *RXRA* CpG 11 |
| --- | --- | --- | --- | --- | --- | --- | --- | --- | --- |
| *RXRA* CpG 1† | 1 |  |  |  |  |  |  |  |  |
| *RXRA* CpG 2 | 0.89 | 1 |  |  |  |  |  |  |  |
| *RXRA* CpG 3† | 0.93 | 0.93 | 1 |  |  |  |  |  |  |
| *RXRA* CpG 4 | 0.91 | 0.9462 | 0.91 | 1 |  |  |  |  |  |
| *RXRA* CpG 5 | 0.90 | 0.9202 | 0.89 | 0.9464 | 1 |  |  |  |  |
| *RXRA* CpG 8 | 0.69 | 0.7687 | 0.70 | 0.7715 | 0.7566 | 1 |  |  |  |
| *RXRA* CpG 9 | 0.78 | 0.8094 | 0.77 | 0.8182 | 0.8176 | 0.788 | 1 |  |  |
| *RXRA* CpG 10 | 0.78 | 0.8257 | 0.77 | 0.846 | 0.8312 | 0.8532 | 0.899 | 1 |  |
| *RXRA* CpG 11 | 0.79 | 0.8207 | 0.78 | 0.8451 | 0.835 | 0.8283 | 0.886 | 0.9345 | 1 |
| *RXRA* CpG 12 | 0.57 | 0.6091 | 0.54 | 0.6578 | 0.6413 | 0.8265 | 0.6286 | 0.738 | 0.7297 |
| † Spearman correlation used for CpG1 and CpG3 | |  |  |  |  |  |  |  |  |

**Online Supplementary Table 3:** *RXRA* Median Absolute deviation scores

| **Cluster** | **CpG site** | **MAD score** |
| --- | --- | --- |
| CpG 1-5 | 1 | 5.26 |
|  | 2 | 4.92 |
|  | 3 | 5.35 |
|  | 4 | 5.03 |
|  | **5** | **5.41** |
| CpG 8-11 | 8 | 4.16 |
|  | 9 | 4.58 |
|  | 10 | 4.25 |
|  | **11** | **4.66** |
| CpG 12 | **12** | **3.98** |

**Online Supplementary Table 4:** *RXRA* DNA methylation in cholecalciferol 1000 IU/day supplemented^a^ and placebo^b^ groups at all CpG sites studied. p-values < 0.05 are in bold. q-values were obtained using the Simes method.

| **CpG** | **n** | **% methylation Cholecalciferol 1000 IU/day^a^** | **% methylation**  **Placebo^b^** | **Mean difference % methylation ^(a-b)^** | **95%CI** | **p difference** | **q value** |
| --- | --- | --- | --- | --- | --- | --- | --- |
| *RXRA* CpG 1† | 446 | 31.4 (27.1,37.8) | 32.3 (27.9,40.6) | -0.17 | -0.35, 0.02 | 0.1 | 0.183 |
| *RXRA* CpG 2 | 449 | 54.6 (7.5) | 56.5 (8.4) | **-1.86*** | **-3.34, -0.37** | **0.01** | 0.100 |
| *RXRA* CpG 3† | 450 | 33.9 (29.7,40.7) | 35.8 (31.0,42.5) | **-0.19*** | **-0.37, -0.008** | **0.04** | 0.100 |
| *RXRA* CpG 4 | 444 | 49.8 (8.0) | 51.5 (9.3) | **-1.70*** | **-3.32, -0.08** | **0.04** | 0.100 |
| *RXRA* CpG 5 | 447 | 46.7 (8.2) | 48.7 (9.7) | **-1.98*** | **-3.65, -0.32** | **0.02** | 0.100 |
| *RXRA* CpG 8 | 449 | 62.7 (5.9) | 63.6 (6.5) | -0.92 | -2.07, 0.22 | 0.11 | 0.183 |
| *RXRA* CpG 9 | 448 | 51.1 (7.5) | 52.0 (8.5) | -0.95 | -2.44, 0.53 | 0.21 | 0.233 |
| *RXRA* CpG 10 | 447 | 65.1 (6.8) | 66.0 (7.5) | -0.88 | -2.20, 0.45 | 0.19 | 0.233 |
| *RXRA* CpG 11 | 446 | 58.3 (7.5) | 58.9 (8.1) | -0.67 | -2.12, 0.78 | 0.36 | 0.360 |
| *RXRA* CpG 12 | 446 | 66.1 (5.8) | 66.9 (6.6) | -0.84 | -1.99, 0.31 | 0.15 | 0.214 |

**Online Supplementary Table 5:** Relationships between perinatal methylation in umbilical cord at CpG sites within the *RXRA* region of interest and bone outcomes at birth (measured by DXA, whole body minus head), stratified by placebo and 1000IU / day cholecalciferol supplemented groups. Associations are adjusted for sex. β coefficients and 95% CIs have been multiplied by 10 and therefore represent the change associated with a 10% increase in methylation. p-values < 0.05 are in bold.

|  | **BA, (cm^2^)** | | | | **BMC, (g)** | | | | **aBMD, (g/cm^2^ )** | | | |
| --- | --- | --- | --- | --- | --- | --- | --- | --- | --- | --- | --- | --- |
|  | **Placebo** | | **1000 IU/d** | | **Placebo** | | **1000 IU/d** | | **Placebo** | | **1000 IU/d** | |
|  | β (95% CI) | p | β (95% CI) | p | β (95% CI) | p | β (95% CI) | p | β (95% CI) | p | β (95% CI) | p |
| *RXRA* CpG  5 | 5.22 (-0.18, 10.62) | 0.06 | 2.42(-3.78,8.61) | 0.44 | **1.75 (0.17, 3.33)** | **0.03** | 1.08( -0.93, 3.08) | 0.29 | 0.00(-0.00, 0.01) | 0.10 | 0.00(-0.00, 0.01) | 0.27 |
| *RXRA* CpG 11 | **6.96 (0.09, 13.82)** | **0.05** | -1.74( -8.17, 4.69) | 0.59 | **2.34 (0.32, 4.37)** | **0.02** | -0.78( -2.87, 1.31) | 0.46 | 0.00(-0.00, 0.01) | 0.08 | -0.00(-0.01, 0.00) | 0.46 |
| *RXRA* CpG 12 | 7.76 (-0.77, 16.28) | 0.07 | -3.29( -11.86, 5.27) | 0.45 | 2.05( -0.47, 4.57) | 0.11 | -1.18(-3.96, 1.60) | 0.41 | 0.00(-0.00, 0.01) | 0.46 | -0.00(-0.01, 0.00) | 0.51 |

(BA: bone area; BMC: bone mineral content; aBMD: areal bone mineral density)

**Online Supplementary Figure 1:** Location of CpG dinucleotides in the *RXRA* region. Region of interest: (chr9: 137215735- 137216064). (Human genome hg19/GRCh37 build). Local gene layout is shown with relevant distances marked in base-pairs (bp) from the transcriptional start site (TSS) of *RXRA*. The CpG dinucleotides of interest are marked on an annotated primary sequence.


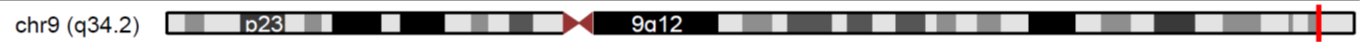

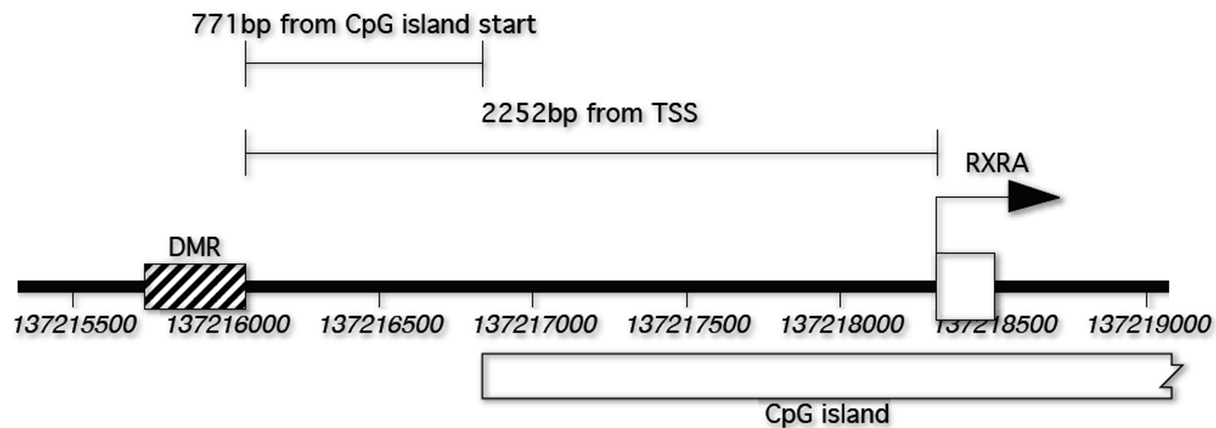

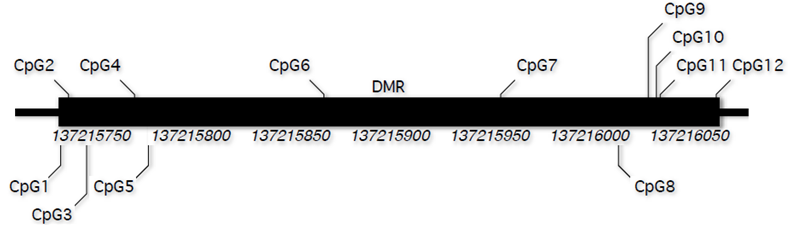


**Online Supplementary Figure 2:** UCSC- Human Genome Reference Sequence annotation for the *RXRA* region, Feb. 2009 (GRCh37/hg19) Assembly. The *RXRA* DMR is annotated in purple. DNase1 hypersensitivity (HS) clusters are displayed across 125 cell types, with examples of DNase1 HS peaks demonstrated in chorion and osteoblast cells. Enrichment of Transcription Factor binding sites (Transcription Factor CHIP-seq (161 factors) are outlined, with an example of peaks overlying the *RXRA* DMR in MCF-7 cells. Enhancer related mono-methylation of lysine 4 of the H3 histone protein (H3K4me1) are demonstrated. Genome segmentations from ENCODE across a variety of cells demonstrate the following predicted functional regions: in red, promoter regions; orange, enhancers; yellow, weak enhancers or open chromatin cis regulatory elements; blue, CTCF transcriptional repressor enriched element; grey, predicted repressed regions. A yellow weak enhancer region is shown in the region of the *RXRA* DMR.

**Online Supplementary Methods: Human Placental Data**

**Placental samples:** The study was conducted according to the guidelines in the Declaration of Helsinki, and the Southampton and South West Hampshire Research Ethics Committee approved all procedures (REC 11/sc/0323). Written informed consent was obtained from all participating women.

Placentas (n = 6) were collected from healthy term pregnancies, not within the MAVIDOS trial, within 30 min of delivery. Placental villous tissue fragments were cultured at 37˚C for 8 h in Tyrode’s buffer containing 0.7 mM albumin with or without 20 µM 25-hydroxyvitamin D [25(OH)D], and then snap frozen and stored at -80°C.

**DNA Methylation:** DNA was extracted from placental samples using the DNeasy Blood & Tissue Kit (Qiagen, UK) according to manufacturer’s instructions. DNA methylation was measured using the Illumina EPIC 850K array and altered CpGs with Vitamin D treatment were identified using a Wilcoxon signed-rank test (p < 0.05).

**RNA Sequencing:** RNA was extracted from placental samples using the miRNeasy mini kit (Qiagen, UK) according to manufacturer’s instructions. Stranded RNA sequencing was carried out by Expression Analysis (Durham, North Carolina, USA) using HiSeq 2x50bp paired-end sequencing on an Illumina platform. Analysis was performed by Expression Analysis to identify the differentially expressed genes following 25(OH)D treatment (p < 0.05), using their in-house developed RNA-Seq bioinformatics pipeline (version 9) which uses a variety of internally developed and open source programs (<https://expressionanalysis.github.io/ea-utils/>).

**Online Supplementary Table 6**

| **CpG** | **Distance from transcriptional start site (bases)** | **Human genome 19 coordinates** | **% difference in methylation**  **(cholecalciferol treatment - no cholecalciferol treatment)** | **P-value** |
| --- | --- | --- | --- | --- |
| cg15266275 | 44772 | 137263193 | -1.89 | 0.01 |
| cg13689699 | 6890 | 137225311 | 1.41 | 0.01 |
| cg14051721 | 82888 | 137301309 | -2.67 | 0.02 |
| cg02059519 | 32514 | 137250935 | -0.80 | 0.04 |
| cg09800519 | -135 | 137218286 | 1.10 | 0.04 |
| cg10984912 | 79935 | 137298356 | -1.26 | 0.02 |

Comparison between *RXRA* methylation at CpG sites in proximity to the *RXRA* gene in human placental villous tissue fragments cultured with or without 20 µM cholecalciferol (n=6). Region: Chromosome 9: 137218286- 1137301309, Human genome hg19/GRCh37 build.
